# Granulostasis: Protein Quality Control of RNP Granules

**DOI:** 10.3389/fnmol.2017.00084

**Published:** 2017-03-27

**Authors:** Simon Alberti, Daniel Mateju, Laura Mediani, Serena Carra

**Affiliations:** ^1^Alberti Lab, Max Planck Institute of Molecular Cell Biology and GeneticsDresden, Germany; ^2^Department of Biomedical, Metabolic and Neural Sciences, Center for Neuroscience and Nanotechnology, University of Modena and Reggio EmiliaModena, Italy

**Keywords:** molecular chaperone complexes, protein homeostasis, RNA homeostasis, stress granules, phase separation, age-related neurodegenerative diseases, amyotrophic lateral sclerosis

## Abstract

Ribonucleoprotein (RNP) granules transport, store, or degrade messenger RNAs, thereby indirectly regulating protein synthesis. Normally, RNP granules are highly dynamic compartments. However, because of aging or severe environmental stress, RNP granules, in particular stress granules (SGs), convert into solid, aggregate-like inclusions. There is increasing evidence that such RNA-protein inclusions are associated with several age-related neurodegenerative diseases, such as amyotrophic lateral sclerosis (ALS), fronto-temporal dementia (FTD) and Alzheimer’s disease (AD). Thus, understanding what triggers the conversion of RNP granules into aggregates and identifying the cellular players that control RNP granules will be critical to develop treatments for these diseases. In this review article, we discuss recent insight into RNP and SG formation. More specifically, we examine the evidence for liquid-liquid phase separation (LLPS) as an organizing principle of RNP granules and the role of aggregation-prone RNA-binding proteins (RBPs) in this process. We further discuss recent findings that liquid-like SGs can sequester misfolded proteins, which promote an aberrant conversion of liquid SGs into solid aggregates. Importantly, very recent studies show that a specific protein quality control (PQC) process prevents the accumulation of misfolding-prone proteins in SGs and, by doing so, maintains the dynamic state of SGs. This quality control process has been referred to as granulostasis and it relies on the specific action of the HSPB8-BAG3-HSP70 complex. Additional players such as p97/valosin containing protein (VCP) and other molecular chaperones (e.g., HSPB1) participate, directly or indirectly, in granulostasis, and ensure the timely elimination of defective ribosomal products and other misfolded proteins from SGs. Finally, we discuss recent findings that, in the stress recovery phase, SGs are preferentially disassembled with the assistance of chaperones, and we discuss evidence for a back-up system that targets aberrant SGs to the aggresome for autophagy-mediated clearance. Altogether the findings discussed here provide evidence for an intricate network of interactions between RNP granules and various components of the PQC machinery. Molecular chaperones in particular are emerging as key players that control the composition and dynamics of RNP granules, which may be important to protect against age-related diseases.

## Introduction

Ribonucleoprotein (RNP) granules are large RNP particles. Examples are processing bodies (PBs) and stress granules (SGs) in the cytoplasm and neuronal transport granules in the axons and dendrites of neurons. These RNP granules have diverse functions in transporting, storing, or degrading RNAs (Buchan and Parker, 2009). Evidence is now accumulating that RNP granules form through the process of liquid-liquid phase separation (LLPS; Brangwynne et al., 2009; Li et al., 2012; Hyman et al., 2014). For phase separation to occur, two components are necessary: RNA-binding proteins (RBPs) and RNAs. An initially homogenous solution of these two components can demix into two co-existing phases, a dense phase that is enriched for protein and RNA and a dilute phase, which then stably co-exist. The dense phase behaves as a compartment that allows selective access of certain proteins and RNAs, but not others (Hyman et al., 2014; Banani et al., 2016; Su et al., 2016).

RBPs that are required for RNP granule formation by phase separation have very unusual biophysical and biochemical properties. This makes these proteins prone to misfold and aggregate, thus creating a need to tightly control them. In agreement, there is increasing evidence of a cross-talk between RNP granules and the protein quality control (PQC) machinery, a system of chaperones and protein degradation factors that monitors the structure of proteins in cells. Moreover, an increasing number of diseases have been associated with defects in protein quality components that specifically affect RBPs and RNP granule formation.

In this review article, we examine emerging ties between PQC and RNP granules. We focus on SGs, which are RNP particles that assemble upon environmental stress (Kedersha and Anderson, 2002). We will first give a general introduction to PQC. We will then discuss how RNP granules are formed and how the process of RNP granule assembly interfaces with PQC. Next, we will examine recent evidence that the quality control system specifically targets SGs, in a process we refer to as granulostasis (Ganassi et al., 2016; Mateju et al., 2017). Finally, we discuss emerging evidence that deregulated granulostasis and SG malfunction cause many neuromuscular and neurodegenerative diseases.

## Proteostasis: General Control of Protein Folding and Misfolding

Most proteins, including RBPs, need to fold into a well-defined three-dimensional structure to properly perform their functions. Although the information for folding into a three-dimensional structure is encoded in the primary sequence of a protein, folding, especially for large multi-domain proteins, often fails (Hartl et al., 2011). Acquiring the native state is particularly challenging within the cellular environment, for several reasons. First, the cytoplasm is a highly crowded and polydisperse environment, which favors promiscuous interactions between exposed hydrophobic regions of newly synthetized proteins. Second, a substantial portion of newly made proteins acquire their native conformations only after association with their specific binding partners. Third, hundreds of proteins within the cell exist in concentrations where they are barely soluble; these so-called supersaturated proteins constitute a metastable sub-proteome that is constantly at risk of irreversible aggregation (Ciryam et al., 2015). Fourth, cells are frequently exposed to proteotoxic conditions and such conditions make the correct folding of proteins and the preservation of their native structures even more difficult. Finally, genetic mutations can increase the propensity of a protein to misfold and aggregate, putting a heavy burden on the protein synthesis machinery (Labbadia and Morimoto, 2015).

Maintaining protein homeostasis is crucial for cellular and organismal health and survival. Failure to correctly handle misfolding-prone proteins can lead to the formation of cytotoxic aggregates and neurodegenerative diseases (Ciryam et al., 2015). To counteract the detrimental effect of aberrant protein folds, cells have evolved a complex system that monitors and assists protein folding. Correct protein folding or proteostasis is ensured by the PQC system, an integrated network of molecular chaperones, co-chaperones and two degradative systems, namely the ubiquitin-proteasome system (UPS) and autophagy (Hartl et al., 2011; Labbadia and Morimoto, 2015).

## Molecular Chaperones

Molecular chaperones transiently interact with and stabilize an unfolded protein in order to facilitate its folding into the native conformation (Hartl et al., 2011). Chaperones assist the folding of proteins, already as they emerge from the ribosome and/or when they transiently unfold, thus preventing their irreversible aggregation. When misfolding and aggregation cannot be prevented (e.g., due to genetic mutations that destabilize protein conformation or decrease protein solubility), molecular chaperones can also promote the targeting of misfolded proteins for degradation. Because of their fundamental function in maintaining a healthy proteome, it is not surprising that molecular chaperones appeared very early in evolution and are present in all kingdoms of life, from archaea to prokarya to eukarya.

Molecular chaperones can be grouped into several classes, and many perform specific functions by residing in certain cell compartments or targeting specific client proteins. A large number of chaperones are also referred to as “stress proteins”, since their expression is induced upon stress, including temperature changes or exposure to oxidative stress or UV light. The best studied molecular chaperones are the Heat Shock Proteins (HSPs), which are grouped into families according to their molecular weight: HSP100, HSP90, HSP70, HSP60 and small HSPs (Lindquist and Craig, 1988). These HSPs all have in common the ability to sense and bind to hydrophobic regions of unfolded/misfolded substrate proteins. However, they prevent protein aggregation with different mechanisms. Small HSPs possess no ATPase activity and are generally considered as “holdases”; they maintain the bound substrate protein in a state competent for further processing in cooperation with other molecular chaperones (Haslbeck et al., 2005). In contrast, ATP-dependent chaperones, such as HSP100, HSP90, HSP70 and HSP60, hydrolyze ATP to cyclically release the unfolded substrate, thereby allowing time for proper folding until the native conformation is reached. Folding can occur in the bulk cytosol (e.g., HSP70 and HSP90) or inside hollow chaperones with a cylindrical shape (HSP60s; Hartl et al., 2011).

**Figure 1:**
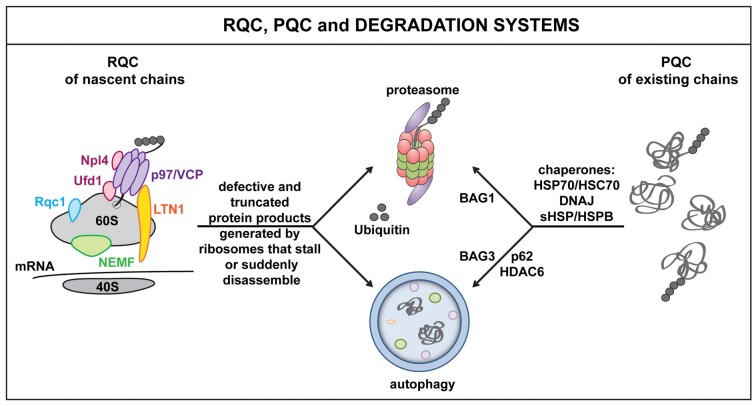
**The ribosome quality control (RQC), protein quality control (PQC) and cellular degradation systems.** Defects at the level of the mRNA as well as defective ribosomal products (DRiPs) activate RQC mechanisms. This leads to the selective elimination of aberrant nascent polypeptides via the proteasome and/or autophagy. Key players of the RQC system include, e.g., LTN1, nuclear export mediator factor (NEMF), valosin containing protein (VCP) and co-factors Npl4 or Ufd1. Similarly, when misfolded proteins accumulate, for example because of proteotoxic stress, molecular chaperones, such as DNAJs and HSPBs, act in concert with HSP70 and specific co-chaperones to target misfolded proteins to the proteasome (BAG1) or autophagy (BAG3) for disposal. Autophagy receptors and adaptor molecules, such as p62 and HDAC6, participate in targeting (poly)ubiquitinated substrates to autophagosomes.

Molecular chaperones often work in concert with a network of other chaperones and co-chaperones. For example, HSP70 switches, in an ATP-dependent manner, between a high- and low-affinity state for unfolded substrates. Chaperones like the DNAJs/HSP40s accelerate the hydrolysis of ATP, thereby facilitating the binding of HSP70 to its clients. HSP70 also binds to nucleotide exchange factors (NEFs), including the BAG proteins; these catalyze the dissociation of ADP from HSP70, ensuring substrate release and chaperone recycling. These factors and co-chaperones can also promote targeting of HSP70-bound client to degradation systems, such as the proteasome or autophagy (Kampinga and Craig, 2010). Alternatively, the bound clients can be transferred to other chaperone machineries, like HSP90 or HSP60, to complete protein maturation (Hartl et al., 2011; Figure 1).

Molecular chaperones constitute a very powerful arm of the PQC system. Chaperones recognize aberrant protein folds and keep the cell free of toxic protein species by promoting their refolding or degradation. Importantly, most of the aberrant protein folds in a cell are produced at the ribosome during protein synthesis (Schubert et al., 2000). For this reason, the ribosome has evolved a very sophisticated chaperone and PQC machinery.

## Protein Quality Control at the Ribosome

Errors are intrinsic to the mRNA translation process and can lead to the production of defective polypeptides that are both aggregation-prone and potentially harmful to the cell. The production of so-called defective ribosomal products (DRiPs) can be a consequence of misincorporation of amino acids, premature translation termination, damaged mRNAs or DNA mutations (Schubert et al., 2000). Therefore, to avoid the accumulation of potentially harmful defective translation products, cells evolved sophisticated PQC systems that operate right at the ribosome.

One example are specific molecular chaperones that assist the cotranslational folding process of newly produced polypeptides. These molecular chaperones immediately interact with nascent polypeptides when they exit from the ribosome. Specialized types of HSP70s and HSP40 co-chaperones are part of the ribosome-nascent peptide complex that facilitates the cotranslational folding process (Beckmann et al., 1990; Nelson et al., 1992; Wang et al., 2013; Gloge et al., 2014). This cotranslational quality control system senses the folding state of these nascent polypeptides during translation and, when it detects defects, recruits degradation factors to clear defective and potentially toxic species.

In addition, cells also evolved a ribosome-associated quality control system (RQC), which senses defects at the level of the mRNA, rather than on the level of the polypeptide (Brandman et al., 2012; Cherkasov et al., 2013). In fact, damaged mRNAs, high amounts of mRNA secondary structures, the presence of repeated sequences, including the poly(A) tail, as well as deficiencies of specific amino acids or tRNAs can all lead to translational stalling; this alerts the RQC and result in the targeting of the nascent polypeptide for degradation. The key RQC players include the E3 ligase Listerin (Ltn1), the nuclear export mediator factor (NEMF/Rqc2/Tae), Rqc1, the nascent polypeptide associated complex (NAC), the AAA+ ATPase valosin containing protein (VCP/Cdc48) and its co-factors Npl4 and Ufd (Bengtson and Joazeiro, 2010; Brandman et al., 2012; Defenouillere et al., 2013; Shao et al., 2015; Yonashiro et al., 2016). These proteins physically associate with the 60S ribosome and orchestrate a series of events that promote the disassembly of the stalled translation complex and the recycling or degradation of ribosomal subunits, mRNAs and nascent polypeptides (Figure 1).

In addition, the defective nascent polypeptide is marked with specific post-translational modifications (PTMs) that dictate the polypeptide’s fate. Polyubiquitination, for example, promotes VCP-assisted targeting of DRiPs to the proteasome or autophagy machinery for disposal (Verma et al., 2013). In contrast, addition of a C-terminal Alanine-Threonine Tail (CAT tail), or CATylation, which is operated by NEMF, makes stalled proteins insoluble and prone to aggregate. CATylation and protein aggregation also activate the expression of canonical chaperones, including HSPs, via the master regulator HSF1, thereby enhancing the chaperone capacity of the cells, likely in an attempt to preserve proteostasis under conditions of translational stress (Brandman and Hegde, 2016; Choe et al., 2016; Figure 1).

Collectively, the cotranslational quality control, the RQC and the post-translational quality control pathways, in concert with the disposal machineries, protect the proteome by ensuring the clearance of potentially harmful DRiPs. Protein disposal by degradation often seems to be the preferred way of inactivating potentially harmful polypeptides.

## Disposal Systems: The Proteasome and Autophagy Pathways

In mammalian cells, two main degradative pathways are responsible for the clearance of misfolded proteins: the UPS and the autophagy machinery (Ciechanover, 2005). The UPS is a highly specific degradative pathway that is normally involved in the degradation of short-lived nuclear and cytosolic proteins carrying ubiquitin chains (Glickman and Ciechanover, 2002; Ravid and Hochstrasser, 2008). Degradation of proteins through the UPS requires the concerted action of enzymes, chaperones and co-chaperones. The linkage of ubiquitin chains to the target protein is mediated by an enzymatic cascade, comprising an E1 ubiquitin-activating enzyme, E2 ubiquitin-conjugating enzyme(s) and E3 ubiquitin ligase(s). The polyubiquitinated proteins are then recognized and processed by the 26S proteasome, a barrel-shaped multicatalytic complex consisting of two major parts: the 19S regulatory component and the 20S core catalytic component. The 19S complex recognizes the substrate, unfolds it via the ATPase subunits, and removes the ubiquitin tags. Then, the deubiquitinated substrate enters the catalytic barrel of the 20S proteasome, where it is degraded (Glickman and Ciechanover, 2002; Ravid and Hochstrasser, 2008).

The disposal of proteins by the UPS often requires the cooperation of chaperones and co-chaperones. One example of a complex that targets client proteins to the proteasome is the HSP70-BAG1-CHIP complex. BAG1 is one of the six members of the BAG (Bcl-2 associated athanogene) family of proteins (Takayama and Reed, 2001); it shuttles misfolded proteins to the proteasome and acts as a NEF of HSP70. CHIP, on the other hand, is an E3 ligase that interacts, via its tetratricopeptide repeat (TRP) domain, with chaperones such as HSC70, HSP70 and HSP90; CHIP suppresses the ATPase activity of HSC70/HSP70 and directs the substrates to the proteasome by tagging them with ubiquitin (Demand et al., 2001; Alberti et al., 2002; Qian et al., 2006). Although the UPS system plays a crucial role in the disposal of misfolded proteins, it can only degrade proteins that can be processed and unfolded via the 19S regulatory particle functions. Aggregated proteins may not fit into the proteasome barrel and are instead degraded via an alternative pathway, referred to as autophagy (Yang and Klionsky, 2009).

Autophagy is a conserved mechanism that acts to maintain cellular nutrient levels and intracellular organelle homeostasis; it also plays an essential role in the removal of potentially harmful aggregated proteins. The deregulation of autophagy has been implicated in a wide range of disorders, including neurodegeneration and cancer (Yang and Klionsky, 2010). Autophagy can be classified into macroautophagy, microautophagy and chaperone-mediated autophagy (CMA; Yang and Klionsky, 2009). Macroautophagy is a multistep process that involves the formation of a double-membraned vesicle, the autophagosome, which is degraded by subsequent fusion with lysosomes (Mizushima et al., 2008). Microautophagy involves the direct sequestration of cytosolic components by invagination of lysosomal membranes. CMA is a highly selective mechanism that degrades soluble cytoplasmic substrates containing a lysosome-targeting motif (Arias and Cuervo, 2011).

Although for a long time macroautophagy (often referred to as autophagy) was considered a non-selective degradation process, recent evidence has uncovered various forms of selective autophagy. Specific autophagy pathways exist to degrade mitochondria (mitophagy), lipid droplets (lipophagy), peroxisomes (pexophagy), ribosomes (ribophagy) and protein aggregates (aggrephagy) (Yang and Klionsky, 2009). Specific target recognition is mediated by cargo receptors/adaptors together with the yeast Atg8 or its mammalian homolog LC3, which are ubiquitin-like modifiers that localize to autophagosome membranes (Cohen-Kaplan et al., 2016). Cargo receptors harbor specialized domains that mediate the interaction with target proteins and autophagy machinery. p62/SQSTM1 (sequestosome 1) and NBR1 (neighbor of BRCA1 gene 1), for example, possess a UBA domain, which allows them to bind ubiquitinated substrates, and a LIR domain, which targets the cargo to autophagosomes by interacting with LC3 (Kirkin et al., 2009; Lamark et al., 2009). Importantly, p62 has a preference for K63-based ubiquitin chains, which function as targeting signal for autophagy rather than for proteasomal degradation (Olzmann et al., 2007; Tan et al., 2008). p62 also interacts with the HSPB8-BAG3-HSP70 chaperone complex and together they sequester misfolded ubiquitinated proteins for selective autophagy (Minoia et al., 2014). Cargo receptors such as p62 thereby ensure the clearance of aggregated proteins that would otherwise accumulate in cells, with potentially toxic consequences. Indeed, p62 and ubiquitin are frequently found on protein inclusions that accumulate in protein conformation disorders, such as Parkinson’s disease, Huntington’s disease or amyotrophic lateral sclerosis (ALS; Menzies et al., 2015).

Although the proteasome and autophagy systems are distinct pathways for proteins degradation, they are linked by specific players (Pandey et al., 2007; Zhao et al., 2007). Key players of the UPS/autophagy cross-talk include, besides p62, ALFY (autophagy-linked FYVE protein) and HDAC6 (histone deacetylase 6). ALFY interacts with p62 and mediates the selective degradation of ubiquitinated cargoes via autophagy (Clausen et al., 2010; Filimonenko et al., 2010). HDAC6 binds to ubiquitin and the molecular motor dynein and carries misfolded proteins to the microtubule organizing center (MTOC). By doing so, HDAC6 assists the packaging of misfolded proteins into so-called aggresomes, for subsequent storage or degradation via autophagy (Kawaguchi et al., 2003; Lee et al., 2010). Additional factors that promote a cross-talk between the UPS and autophagy machinery are chaperone complexes. For example, the HSPB8-BAG3-HSP70 chaperone complex mediates the selective targeting of chaperone-bound substrates to autophagy in concert with p62 and dynein (Gamerdinger et al., 2011).

More generally, chaperone complexes play important roles in deciding the fate of client proteins, and this decision is often dependent on the overall activity of the autophagy and UPS systems. For example, under conditions of proteasome impairment, the HSPB8-BAG3-HSP70 chaperone complex is upregulated, which then re-routes (poly)ubiquitinated clients to p62 bodies for autophagy-mediated disposal (Minoia et al., 2014). In contrast, upon inhibition of dynein-mediated transport to the MTOC, which is required for efficient aggresome formation and autophagy-mediated degradation, cells preferentially upregulate BAG1. Association of BAG1 with HSP70 then re-routes chaperone clients to the proteasome (Cristofani et al., 2017). Combined these data demonstrate that the UPS, the autophagic machinery and specific chaperone complexes (e.g., BAG1-CHIP-HSP70 and HSPB8-BAG3-HSP70) cooperate to ensure the degradation of abnormal proteins, thereby maintaining a healthy proteome.

An increasing number of studies now suggest that failure of quality control mechanisms causes age-related neurodegenerative diseases, such as ALS and fronto-temporal dementia (FTD). A hallmark of these diseases is that specific proteins called prion-like proteins misfold and form cytotoxic aggregates. But why are these proteins so prone to aggregate and why is the PQC machinery no longer able to keep these proteins under control? As we discuss in the next section, prion-like proteins have very unusual biophysical properties, which are required for these proteins to perform their physiological function as organizers of intracellular compartments. However, the ability to form compartments becomes detrimental with increasing age, because aging impairs the proteostasis machinery of cells and thus increases the probability that prion-like proteins will form irreversible aggregates.

## Prion-Like Proteins, Cell Organization and Age-Related Diseases

Cellular functionality depends on intracellular organization and compartmentalization. There are two types of compartments in eukaryotic cells: membrane-bound and membrane-less compartments. For membrane-bound compartments it is easy to understand how they perform their function: they separate biomolecules through lipid bilayers. However, how membrane-less compartments separate biomolecules has only recently become clear. Evidence now suggests that membrane-less compartments form through the process of LLPS (Brangwynne et al., 2009; Li et al., 2012; Hyman et al., 2014). In LLPS an initially homogenous solution of components becomes supersaturated and then demixes into two phases that then stably co-exist. One of these phases has properties of a dense liquid that continuously exchanges material with the surrounding environment. The interface of the dense liquid forms a compartment boundary, which controls the exchange of material with the surrounding milieu. Examples of membrane-less compartments are RNP granules (Brangwynne et al., 2009), centrosomes (Woodruff et al., 2016) and nucleoli (Brangwynne et al., 2011).

Membrane-bound compartments require lipids for compartment formation. But which macromolecules are required for the formation of membrane-less compartments? Recent findings suggest that the formation of membrane-less compartments such as RNP granules requires RNA and a special class of multivalent proteins (Lin et al., 2015; Molliex et al., 2015; Patel et al., 2015). These proteins often contain intrinsically disordered regions that are characterized by low complexity sequence (LCS). Low complexity regions only contain a subset of the 20 possible amino acids and they often have a repetitive nature.

There is a special class of low complexity proteins that contain mostly polar amino acids, such as glutamine, asparagine and serine. These proteins are often referred to as prion-like proteins, because their composition is similar to proteins in budding yeast that form heritable aggregates or prions (Alberti et al., 2009; King et al., 2012; Malinovska et al., 2013). There is now increasing evidence that prion-like proteins are highly aggregation-prone and cause several diseases when mutated (Li et al., 2013; Taylor et al., 2016). One example is the RNA-binding protein TDP-43, which, when mutated in the C-terminal prion-like region, aggregates in the cytoplasm and causes ALS and related diseases. Another example is the RNA-binding protein FUS, which is associated with some of the most aggressive forms of ALS and FTD. Mutations in FUS are often found in the C-terminal nuclear localization sequence, which changes the localization of the protein and leads to the formation of cytotoxic aggregates in the cytoplasm (Lagier-Tourenne et al., 2010).

Studies in the last couple of years have begun to unravel the molecular function of prion-like low complexity regions. Most of the evidence comes from experiments with the two prion-like proteins FUS and hnRNPA1. These experiments suggest that the LCS of FUS and hnRNPA1 are required for the formation of compartments by LLPS (Molliex et al., 2015; Patel et al., 2015). The polymer nature of the LCS and their ability to undergo many weak interactions allow these proteins to phase separate and form liquid droplets. Prion-like proteins such as FUS need to be in a state of supersaturation in order to phase separate. In agreement, the cellular concentration of these proteins is under tight control, and the solubility and self-interaction of prion-like proteins are regulated by PTMs (Wippich et al., 2013; Nott et al., 2015).

**Figure 2:**
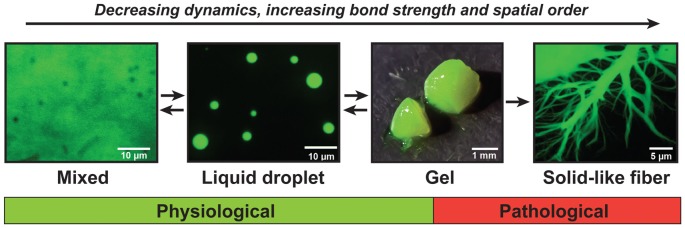
**The continuum model of prion-like proteins.** FUS and other prion-like proteins can adopt a range of different material states. When supersaturated, FUS demixes from a solution of FUS molecules and assembles into liquid-like droplets. These liquid droplets are unstable and will convert with time into gels and solid-like fibers. The liquid-like state is required for the formation of physiological compartments in the nucleus and cytoplasm of cells, whereas the gel and fiber states have been associated with pathological conditions.

Recent findings show that, when large numbers of FUS molecules interact, they can adopt a range of different material states (Murakami et al., 2015; Patel et al., 2015). FUS first forms dense liquids in *in vitro* reconstitution experiments, but these liquids are metastable and mature into gels and fibers that are reminiscent of pathological aggregates found in patients (Patel et al., 2015). This process has been referred to as molecular aging, and the ability to adopt different states is now known as the continuum model (Figure 2; Alberti and Hyman, 2016; Bergeron-Sandoval et al., 2016). Importantly, the molecular aging of FUS is accelerated by mutations that have been identified in ALS and FTD patients.

Thus, in conclusion, there is a group of proteins such as FUS and TDP-43 whose function is to form intracellular compartments. There are more than 100 proteins with similar sequence compositions as FUS and TDP-43, suggesting that many additional compartment-forming proteins exist that may be involved in diseases (King et al., 2012; Malinovska et al., 2013). However, for most of these proteins we do not know their physiological functions, nor do we know whether they are involved in pathologies. However, there is one membrane-less compartment known as SGs that contains many disease-associated prion-like proteins. It has been proposed that aberrant SG behavior underlies age-related diseases such as ALS and FTD. Thus, in the following, we will discuss the link between SGs, prion-like proteins and PQC.

## Stress Granules: Function, Composition and Assembly

Cells are frequently exposed to stress, such as high temperature, UV light, oxidative stress, starvation or viral infection (Morimoto, 2011). To cope with such stressful conditions, cells have evolved mechanisms to conserve energy and protect macromolecules. One way to achieve these goals is to shut down translation of house-keeping genes and prioritize the synthesis of enzymes and chaperones required for stress adaptation. Translation inhibition is often induced via phosphorylation of the initiation factor 2 alpha (eIF2alpha) and followed by polyribosome disassembly. This in turn leads to the release of translation initiation factors, ribosomal subunits and mRNAs that are coated with RBPs. The released mRNA-RBP complexes are then packaged into membrane-less RNP compartments that are called SGs. These SGs sequester the released mRNAs and RBPs and keep them silent and protected from degradation until the stress subsides (Anderson and Kedersha, 2002).

Polyribosome disassembly not only leads to the release of mRNAs and translation factors but also of newly synthesized polypeptides. These polypeptides fold into their native structure with the assistance of chaperones or they associate with other proteins to form native complexes. However, a surprisingly high number of released polypeptides are defective. There are estimates that between 6% and 30% of all newly synthesized proteins are DRiPs and are very rapidly degraded by the UPS (Schubert et al., 2000; Qian et al., 2006). These DRiPs can arise from premature translation termination, faulty mRNAs or ribosomal frameshifting (Yewdell, 2011). It is very likely that DRiP formation is strongly increased under stress conditions, because they generally lead to cotranslational misfolding and premature termination events (Lelouard et al., 2004; Szeto et al., 2006).

SGs are highly dynamic structures, and proteins and RNAs that localize in SGs often have only very short residence times in SGs. The nucleation of SGs also occurs rapidly via self-assembly of RBPs that contain prion-like low complexity domains, including e.g., TIA-1, G3BP, FUS, hnRNPA1 (Gilks et al., 2004; Cushman et al., 2010). Increasing evidence suggests that these proteins have the ability to phase separate and form SGs through LLPS (Kroschwald et al., 2015; Molliex et al., 2015; Patel et al., 2015). Although SGs clearly have properties of dense liquids (Molliex et al., 2015; Patel et al., 2015), there also is evidence that they contain solid-like “cores” (Jain et al., 2016; Wheeler et al., 2016). It has been postulated that these cores are nucleating RNPs that initiate the assembly of SGs (Wheeler et al., 2016). However, it remains a possibility that the cores are liquid-like initially, but then quickly harden through a molecular aging process.

SGs not only contain RBPs and RNAs but also additional factors, such as 40S ribosomal subunits, eIF2, eIF3, eIF4E, eIF4G, eIF4A, eIF4B, poly(A)-binding protein (PABP). Depending on experimental conditions, they also contain RNA helicases and factors involved in cell signaling, such as mTORC1 (Buchan and Parker, 2009). Sequestration of mTORC1 inside SGs allows the control of mTORC1 signaling during stress and is mediated by the dual specificity tyrosine-phosphorylation-regulated kinase 3 (DYRK3; Wippich et al., 2013). Importantly, DYRK3 partitioning into SGs is regulated by its own kinase activity.

In fact, growing evidence now suggests that PTMs of SG components influence the formation, composition and function of SGs. These PTMs comprise phosphorylation, acetylation, O-glycosylation and methylation reactions (Gallouzi et al., 1998; Tourrière et al., 2001, 2003; Schmidlin et al., 2004; Stoecklin et al., 2004; De Leeuw et al., 2007; Kwon et al., 2007; Goulet et al., 2008; Ohn et al., 2008). For example, phosphorylation regulates the accumulation of TTP, G3BP and BRF1 inside SGs (Gallouzi et al., 1998; Tourrière et al., 2001, 2003; Schmidlin et al., 2004; Stoecklin et al., 2004). Moreover, addition of O-Glc-NAc sugar moieties to RBPs enhances SG formation (Ohn et al., 2008). Regulation by reversible PTMs ensures that SGs are formed rapidly in response to stress, but also quickly dissolve again when the stress conditions subside. Therefore, a better understanding of how PTMs control SG dynamics, and thus protein production and cell signaling, will be an important future objective.

## Stress Granules Recruit PQC Factors and Autophagy Machinery

Early studies indicated that SGs also contain factors involved in PQC, including chaperones, components of the UPS and autophagy factors. This suggested that there may be close ties between the SG response and the PQC system. One of the first PQC components shown to accumulate in heat-inducible SGs was the small HSP HSPB1/HSP27 (Collier and Schlesinger, 1986; Scharf et al., 1998; Kedersha et al., 1999). Another small HSP, HSPB8/HSP22, was later found to localize to SGs that form upon proteotoxic stress (Ganassi et al., 2016). Additional PQC players that reside in SGs have been identified; this includes, among others, ubiquitin, HDAC6 and VCP/p97 (Kawaguchi et al., 2003; Kitami et al., 2006; Kwon et al., 2007; Buchan et al., 2013). VCP/p97 is an ubiquitin-binding protein involved in degrading ubiquitylated substrates; it is also a key component of several degradation pathways, such as ERAD (ER-associated degradation) and co-translational protein degradation (Meyer and Weihl, 2014). HDAC6 recognizes misfolded proteins and assists aggresome formation at the centrosome via its ability to associate with the motor protein dynein (Kawaguchi et al., 2003). Together, these findings demonstrate that many different PQC components accumulate in SGs. But why are these factors recruited to SGs? Which client proteins do they target? And what is the fate of these client proteins?

An important study in 2014 by the Parker and Taylor labs provided evidence that SGs can be degraded by autophagy and that VCP/p97 plays a key role in this process (Buchan et al., 2013). Further support for a role of autophagy came from the finding that the autophagy receptor p62 accumulates specifically in SGs (Matus et al., 2014). This has led to the hypothesis that SGs are directly targeted and cleared by autophagy machinery. However, two questions remained unresolved: is autophagy the preferred pathway of SG clearance? And is autophagy-mediated SG disposal specific or not?

Two recent studies now shed light on these unresolved questions (Ganassi et al., 2016; Mateju et al., 2017). These studies provided evidence for two different types of SGs: physiological SGs and aberrant SGs. Physiological SGs are free of misfold proteins and PQC factors and consequently not recognized by the autophagy machinery; aberrant SGs instead contain misfolded proteins and these misfolded proteins attract PQC factors and autophagy machinery to SGs. Importantly, a very recent study suggests that aberrant SGs are very abundant in organisms of increasing age (Lechler et al., 2017).

Additional findings suggest that aggresome formation plays a key role in linking SG clearance to autophagy (Mateju et al., 2017). The aggresome is a large protein inclusion that sequesters misfolded proteins in an HDAC6-dependent manner (Kawaguchi et al., 2003; Lee et al., 2010). Aggresome formation is usually turned on in cells where the clearance capacity for misfolded proteins is exceeded. Findings by Mateju et al. (2017) now show that aberrant SGs are targeted by PQC factors and then collected at the centrosome in a microtubule-dependent manner. Moreover, aberrant SGs that are sequestered at the aggresome are selectively degraded by autophagy. In agreement with these recent findings, the SG-localized proteins HSPB1 and HSPB8 have also been shown to localize to the aggresome (Bolhuis and Richter-Landsberg, 2010; Ganassi et al., 2016). Moreover, the SG-localized proteins VCP/p97 and HDAC6 both play a key role in aggresome formation (Ju et al., 2008). Thus, SG and aggresome formation seem to be intimately linked processes, and targeting of SGs to aggresomes may be a specific mechanism to rescue cells from an overload of aberrant SGs.

Further evidence for this model comes from recent findings suggesting that autophagy is not the preferred pathway for SG clearance (Ganassi et al., 2016; Mateju et al., 2017). The great majority of aberrant and physiological SGs disassemble when the stress subsides and the components contained in SGs are recycled. However, if autophagy is not the preferred pathway of SG clearance, how are SGs normally dissolved? As we discuss in the next section, molecular chaperones play essential roles in disassembling and releasing the stored components from SGs, and this specifically affects SGs that have adopted an aberrant composition and behavior.

## Molecular Chaperones Regulate Stress Granule Disassembly

SGs are highly dynamic structures that usually disassemble within a few hours after their formation, even if the stress conditions persist. Indeed, short-term proteasome inhibition induces SG assembly, with a maximal peak* circa* 3 h after treatment, whereas prolonged proteasome inhibition results in SG dissolution, typically 6–8 h after treatment (Mazroui et al., 2007; Seguin et al., 2014). The disassembly of SGs allows the cells to recycle SG components, such as proteins, signaling molecules, mRNAs and 40S ribosomal subunits, which otherwise would have to be synthesized *de novo*. Thus, from an energetic point of view, the storage and release of SG components represents a more attractive solution than protein degradation and *de novo* synthesis.

To ensure a timely SG dissolution, cells require a functional PQC machinery. The ATP-driven chaperone HSP70 appears to act as a master regulator of SG dissolution. Early evidence implicated HSP70 in SG dissolution (Mazroui et al., 2007). However, how exactly HSP70 regulates the dynamics of SGs remained unclear. This has changed with the discovery that HSP70 specifically targets misfolded proteins that accumulate in SGs (Ganassi et al., 2016; Mateju et al., 2017).

As discussed in one of the previous sections, HSP70 plays a pivotal role in recognizing aggregation-prone (misfolded) proteins. Once HSP70 has bound a client protein, it can target it to the main degradative pathways (UPS and autophagy), with the assistance of chaperones and co-chaperones (e.g., DNAJs, BAG1, BAG3). Importantly, proteotoxic stress conditions that induce SGs also lead to the accumulation of misfolded proteins, including DRiPs; therefore, it seems possible that SGs could co-aggregate with misfolded proteins. This was initially demonstrated in yeast and *Drosophila melanogaster* cells, where misfolding-prone model proteins, such as firefly luciferase and VHL (von Hippel-Lindau tumor suppressor), were shown to colocalize with heat shock-induced SGs (Cherkasov et al., 2013; Kroschwald et al., 2015; Walters and Parker, 2015). However, no such phenotype was observed in human cells, indicating that this process may be species-specific.

**Figure 3:**
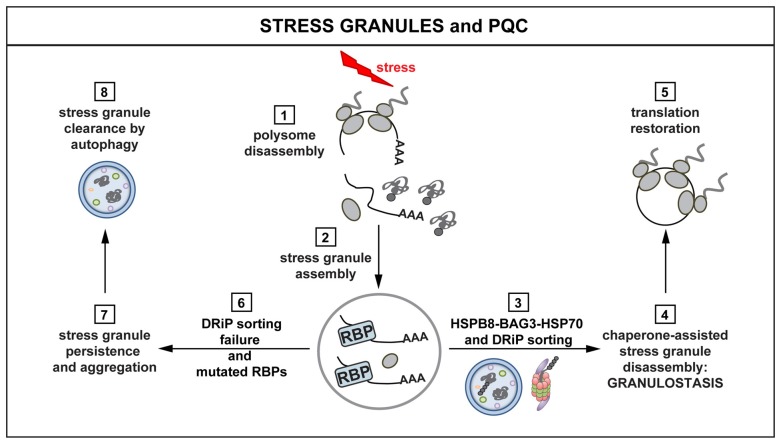
**Interplay between stress granules (SGs) and PQC.** Polysomes disassemble when cells are exposed to stress, releasing mRNAs, ribosomal subunits and DRiPs (1). mRNAs are packaged into SGs (2). The concerted action of the HSPB8-BAG3-HSP70 complex limits the accumulation of misfolded proteins and DRiPs inside SGs (3). The surveillance function of HSPB8-BAG3-HSP70 ensures dynamic SG behavior and disassembly (granulostasis; 4), with subsequent translation restoration (5). Impairment of the HSPB8-BAG3-HSP70 complex and the presence of disease-linked mutations in RBPs leads to the formation of SGs that increasingly accumulate DRiPs (6) and, consequently, become less dynamic and more aggregate-like (7). Persisting SGs can be degraded via autophagy (8). SG persistence affects the ability of the cells to properly restore translation after stress, with potentially harmful effects and loss of cellular viability.

Recent studies indeed suggest that there may be important differences in SG properties across different species. In comparison to mammalian SGs, yeast SGs are more solid-like, and more prone to co-aggregate with misfolded proteins (Kroschwald et al., 2015). Moreover, yeast SGs can be nucleated by misfolded proteins (Cherkasov et al., 2013; Kroschwald et al., 2015). Furthermore, the disassembly of yeast SGs depends on the chaperone Hsp104, which is also required for restoration of mRNA translation in the recovery phase (Cherkasov et al., 2013; Kroschwald et al., 2015). However, mammalian cells lack a cytosolic homolog of Hsp104. Instead, in mammalian cells the HSP70 machinery prevents the co-aggregation of SGs with misfolded proteins such as DRiPs (Ganassi et al., 2016; Mateju et al., 2017; Figure 3). Under severe stress or PQC impairment, SGs co-assemble with misfolded proteins even in mammalian cells.

When DRiPs and other misfolded proteins accumulate inside SGs, the biochemical and dynamic properties of SGs are strongly affected (Ganassi et al., 2016; Mateju et al., 2017). Importantly, aberrant SGs that contain misfolded proteins show decreased dynamics and are resistant to treatment with RNase. This is in line with recent findings showing that SGs are composed of a dynamic, RNase-sensitive shell and a protein-rich, RNase-resistant core (Jain et al., 2016). Thus, one possibility is that DRiPs and other misfolded proteins become enriched inside SGs, and this leads to the formation of aggregate-like (RNase-resistant) core structures inside SGs. This is supported by the finding that isolated core structures contain high amounts of chaperones (Jain et al., 2016). These chaperones could be recruited because of the presence of misfolded proteins in core structures. In agreement, key SG proteins, such as G3BP1, become less dynamic in SGs that contain high levels of misfolded proteins (Mateju et al., 2017). Moreover, SGs that contain misfolded proteins become resistant to RNase, indicating that RNA is no longer required for the structural integrity and that these SGs have converted into protein aggregates. An alternative possibility is that the core structures are solid RNPs that nucleate the formation of SGs. Dissection of this important problem will require a more detailed spatiotemporal analysis of SG formation.

Which chaperones and chaperone factors are required for targeting misfolded proteins in mammalian SGs? Recent findings indicate that this depends on the concerted action of the HSPB8-BAG3-HSP70 complex. This complex ensures targeting of DRiPs to aggresome and degradation pathways, thereby limiting their accumulation inside SGs. This, in turn, helps promoting their dynamic disassembly (Ganassi et al., 2016). More specifically, HSPB8, which is a chaperone holdase, is recruited inside SGs to prevent promiscuous interactions between DRiPs and SG components. When misfolded proteins, such as DRiPs, become entrapped inside SGs, HSPB8 recruits the BAG3-HSP70 subcomplex to sort and process them (Ganassi et al., 2016). This surveillance function at the level of SGs has been referred to as “granulostasis”. Although the HSPB8-BAG3-HSP70 complex plays a pivotal role in granulostasis, other chaperones are recruited as second line of defense, especially when SGs become enriched for additional misfolded proteins at later time points. The small HSP HSPB1 is such a late arriver that could help to prevent conversion of aberrant SGs into irreversible aggregates (Ganassi et al., 2016; Mateju et al., 2017; Figure 3).

The accumulation of misfolded proteins in membrane-less compartments seems to be a more general phenomenon. In fact, a recent study showed that misfolded proteins accumulate in the nucleolus and form a so-called amyloid or A body under heat or acid stress conditions (Audas et al., 2016). Dissolution of protein aggregates in A bodies requires the action of chaperones such as HSP70. Thus, similar chaperone machineries may be working in other membrane-less compartments to regulate and reverse the accumulation of misfolded proteins.

## Emerging Connections Between Stress Granules and Age-Related Diseases

Increasing genetic and experimental evidence supports the interpretation that deregulated SG dynamics and RNA metabolism participate in the pathogenesis of age-related neurodegenerative diseases, including Alzheimer’s disease (AD), ALS, FTD, inclusion body myopathy (IBM) and multisystem proteinopathy (Taylor et al., 2016). First, one of the key signatures of these diseases is the accumulation of cytosolic protein/RNA aggregates. These aggregates are enriched for TDP-43, a nuclear RNA-binding protein that regulates RNA metabolism and is recruited into SGs upon stress (Lagier-Tourenne et al., 2010; Prudlo et al., 2016). Second, several pathogenic mutations in genes encoding SG components have been documented, including mutations in TARDBP, FUS, HNRNPA1, TIA-1, HNRNPDL; many of these disease-linked mutations increase the propensity of affected proteins to convert from a dynamic liquid-like state into amyloid-like fibrils, with consequences for SG dynamics and mRNA metabolism (Hackman et al., 2013; Kim et al., 2013; Klar et al., 2013; Vieira et al., 2014; Lin et al., 2015; Molliex et al., 2015; Murakami et al., 2015; Patel et al., 2015). Third, it is now becoming clear that pathogenic expansions of a hexanucleotide repeat in C9ORF72 are the most common cause of ALS and FTD. C9ORF72 causes the production of toxic dipeptide repeat (DPR) proteins via repeat-associated non-AUG (RAN) translation. These DPRs interact with LCS in RBPs, thus altering the dynamics of membrane-less organelles, such as nucleoli and SGs (Lee et al., 2016). In addition, cells expressing the C9ORF72 repeat expansion form RNA foci that sequester key RBPs involved in RNA metabolism (Lee et al., 2016). The observation that DPRs impair SG dynamics in a similar manner as disease-linked mutations in SG components (e.g., TDP-43, hnRNPA1, FUS) puts further emphasis on the idea that deregulation of RNP granules is a central pathogenic event in many age-related diseases.

There is now clear evidence that mutated or dysfunctional RBPs cause many age-related neurodegenerative diseases, such as ALS and FTD. However, another important group of proteins that have been linked to neurological diseases, including ALS, motor neuropathies and IBM, are molecular chaperones and PQC factors. Studies have identified mutations in genes coding for the molecular chaperone VCP/p97, the autophagy receptor p62, or the chaperones and co-chaperones HSPB1, HSPB8 and BAG3 (Evgrafov et al., 2004; Irobi et al., 2004; Mizuno et al., 2006; Selcen et al., 2009; Johnson et al., 2010; Teyssou et al., 2013; Rea et al., 2014; Ghaoui et al., 2016). The fact that a dysfunctional HSPB8-BAG3-HSP70 or VCP complex results in altered SG composition and dynamics provides further support for a strong interdependence of PQC, SG homeostasis and disease.

Therefore, we propose the following model: on the one hand, mutations in SG components increase their propensity to aggregate; this promotes promiscuous interactions within SGs and increases the tendency of SGs to accumulate misfolded proteins and DRiPs, thus impairing SG functionality (Ganassi et al., 2016). On the other hand, functional impairment of HSPB8-BAG3-HSP70 or VCP cause increased retention of misfolded proteins inside SGs, favoring the conversion of SG components into fibrillary aggregates. Together, these data suggest a strong relationship between PQC and RNA metabolism and further support the idea that molecular imbalances, due to mutations or aging, selectively affect SG biogenesis, which finally leads to a loss of homeostasis and neuronal demise.

This interpretation is supported by recent data linking deregulated SG dynamics with aging. In particular, using *C. elegans* and senescent cell models it has been shown that SG dynamics decrease with aging due to co-aggregation of RBPs with misfolded proteins (Gallouzi, 2009; Lechler et al., 2017; Moujaber et al., 2017). Importantly, in *C. elegans*, the aggregation of SG components is associated with decreased cell fitness and aging, while prevention of RBP aggregation by activation of the heat shock transcription factor HSF-1 and of longevity pathways maintains healthy aging (Lechler et al., 2017). Combined these results provide direct evidence that deregulated aggregation of RBPs is implicated in the aging process and contributes to the development of age-related neurodegenerative diseases. Delayed disassembly and clearance of SGs in aging cells would not only compromise the function of RBPs that become entrapped inside aggregating SGs, but it would also impair the synthesis of many proteins that are required for cell adaptation. Altogether, the insolubilization of critical RBPs and the impaired synthesis of critical cell components would render aged cells more vulnerable, with detrimental consequences for their function and viability.

It is not yet clear whether SG aggregation during aging is due to a failure of the granulostasis machinery. However, the observations that SGs co-aggregate with misfolded proteins and that boosting the heat shock response maintains RBP dynamics strongly suggest that upregulation of specific molecular chaperones may represent a valid approach to combat aging and age-related neurodegenerative diseases. Besides the granulostasis machinery, yeast Hsp104 and the mammalian disaggregase machinery composed of HSP110, HSP70 and HSP40 may represent attractive candidates, whose induction may dissolve aggregated RNPs, thus exerting anti-aging protective effects (Shorter, 2011, 2017; Jackrel et al., 2014). Intriguingly, cooperation between the mammalian disaggregase machinery and human small HSPs has been shown to potentiate the dissolution of amyloid aggregates (Duennwald et al., 2012). This finding opens the possibility that cross-talk between the HSPB8-BAG3-HSP70 granulostasis machinery and the disaggregase machinery might exist and that boosting the latter might represent a powerful tool to prevent the accumulation of aberrant RNP granules, thereby maintaining a state of healthy aging and delaying disease progression.

## Concluding Remarks

The findings discussed here summarize the many mechanisms by which cells cope with proteotoxic stress conditions both as a response to acute external insults or to chronic disease-linked stress; they further highlight how aberrant phase transitions may represent a key triggering event in aging and in a range of neurodegenerative diseases, including ALS and FTD. In particular, this review has emphasized the crucial role of chaperone complexes, with a focus on the HSP70 chaperone machinery. Indeed, chaperones seem to assume a dual functional role in controlling protein homeostasis as well as the composition and function of mRNP SGs, thereby limiting detrimental effects that occur with increasing age.

Potentiation of chaperones and degradative systems have proved very effective in delaying or rescuing neurodegeneration in a number of models of protein conformation diseases, including ALS, AD and polyglutamine diseases (Ravikumar et al., 2002, 2004, 2005; Crippa et al., 2010). In particular, the upregulation of the chaperone HSPB8, or of its *Drosophila melanogaster* homolog (HSP67Bc) was shown to protect against protein aggregation and toxicity in cell and fly models of ALS and polyglutamine diseases and cooperation between HSPB5 and the mammalian disaggregase machinery was very effective in dissolving protein aggregates (Carra et al., 2010; Duennwald et al., 2012; Crippa et al., 2016). Conversely, mutations in small HSPs such as HSPB1, HSPB3, HSPB5, HSPB8 and the co-chaperones DNAJB6 and BAG3 have all been associated with motor neuron and muscular diseases (Vicart et al., 1998; Selcen et al., 2009; Couthouis et al., 2014). This direct association of mutated chaperones with neurological and muscular diseases supports the notion that chaperones play pivotal role in maintaining healthy neurons and muscle cells. The recent implication of the HSPB8-BAG3-HSP70 complex in SG maintenance provides further evidence for a key link between chaperones, mRNA metabolism and cellular health (Walters and Parker, 2015; Ganassi et al., 2016). This is reinforced by the finding that chaperones such as HSPB1 and VCP are regulators of SG composition, dynamism and dissolution/disposal (Buchan et al., 2013; Mateju et al., 2017). Altogether these results suggest that boosting granulostasis may be a very efficient mechanism to rescue both proteostasis and ribostasis in cells.

## Author Contributions

SA, DM, LM and SC equally contributed to the writing of this review.

## Funding

This work was supported by: Agenzia di Ricerca per la Sclerosi Laterale Amiotrofica (AriSLA) Fondation, Ministero dell’Istruzione, dell’Università e della Ricerca (MIUR) and Ministry of Health (Grant No. GR-2011-02347198) and the Max Planck Society. This is an EU Joint Programme—Neurodegenerative Disease Research (JPND) project. The project is supported through the following funding organizations under the aegis of JPND—www.jpnd.eu. This project has received funding from the European Union’s Horizon 2020 research and innovation programme under grant agreement No. 643417. None of the authors of this manuscript have a financial interest related to this work.

## Conflict of Interest Statement

The authors declare that the research was conducted in the absence of any commercial or financial relationships that could be construed as a potential conflict of interest.
